# A machine learning approach to analyse and predict the electric cars scenario: The Italian case

**DOI:** 10.1371/journal.pone.0279040

**Published:** 2023-01-20

**Authors:** Federico Miconi, Giovanna Maria Dimitri

**Affiliations:** Dipartimento di Ingegneria dell’Informazione e Scienze Matematiche (DIISM), Universitá di Siena, Siena, Italy; Nanjing Forestry University, CHINA

## Abstract

The automotive market is experiencing, in recent years, a period of deep transformation. Increasingly stricter rules on pollutant emissions and greater awareness of air quality by consumers are pushing the transport sector towards sustainable mobility. In this historical context, electric cars have been considered the most valid alternative to traditional internal combustion engine cars, thanks to their low polluting potential, with high growth prospects in the coming years. This growth is an important element for companies operating in the electricity sector, since the spread of electric cars is necessarily accompanied by an increasing need of electric charging points, which may impact the electricity distribution network. In this work we proposed a novel application of machine learning methods for the estimation of factors which could impact the distribution of the circulating fleet of electric cars in Italy. We first collected a new dataset from public repository to evaluate the most relevant features impacting the electric cars market. The collected datasets are completely new, and were collected starting from the identification of the main variables that were potentially responsible for the spread of electric cars. Subsequently we distributed a novel designed survey to further investigate such factors on a population sample. Using machine learning models, we could disentangle potentially new interesting information concerning the Italian scenario. We analysed it, in fact, according to different geographical Italian dimensions (national, regional and provincial) and with the final identification of those potential factors that could play a fundamental role in the success and distribution of electric cars mobility. Code and data are available at: https://github.com/GiovannaMariaDimitri/A-machine-learning-approach-to-analyse-and-predict-the-electric-cars-scenario-the-Italian-case.

## 1. Introduction

In the latest years, great attention has been devoted to the problem of climate change and an effort has been put towards the decrease of gas emissions in the atmosphere, especially due to the still excessive presence of internal combustion engine cars. Therefore, the electrification of the road transport sector is considered as a necessary component towards obtaining the so-called “urban decarbonization”.

The diffusion of electric cars in Italy has started, but still some issues are present, and represent an obstacle to its wide spread. Nowadays, hybrid technology is the most followed by users, particularly due to socioeconomic factors such as cost of investment and range anxiety.

Most likely, the solution that could really represent a clear step towards an ever greener mobility is to aim at the diffusion and use of fuel cell vehicles, as highlighted in previous studies [1].

However, the current Italian scenario is still far from achieving the necessary objectives for decarbonization. So far, in fact, Italy has shown a much lower electric vehicle (EV) adoption rate than other European countries.

In [2] the authors highlight the main Italian driver’s preferences towards electric cars. The analysis conducted in the study confirmed that the vehicle attributes, such as purchase price and fuel economy, strongly influence the purchase decision. Moreover, the time spent to charge the vehicle negatively affects the respondents’ utility, while the fast charging network density is not yet perceived as significant [2].

Closely related to the need to recharge the vehicle battery is the driving range factor. This represents, in fact, one of the main characteristics for an electric car, and at the same time one of the most limiting factors for its adoption by consumers. Previous analyses showed that very relevant factors, contributing to the propensity of buying an electric car (ranging from 28% to 68%) over a petrol one, are jointly represented by improvements in the fast charging network, driving range and financial incentives [3]. However, it should be noted that the presence of economic incentives is not enough if the consumer has no environmental concern and willingness-to-pay a premium [4].

In [5] the authors present an interesting work in which the Rome Fiumicino airport is used as a case study for understanding the penetration index of electric vehicles that will access the airport in the years 2025/2030.

Such prediction is in fact of extreme interest for italian electric companies, which deal with the phase of energy distribution to the final consumer, whether it is a private individual or a company, through the distribution network. As a result, all electrical appliances that are daily powered by electricity, receive energy through the distribution network. These certainly include charging points for electric car batteries, both private and public. In particular, the road coverage of charging stations is an essential element for the spread of electric mobility and although it is still considered insufficient, it is destined to grow considerably in the years to come. In this regard, it is important to refer to the estimates contained in the italian PNRR (National Recovery and Resilience Plan), better known as Recovery Plan, which represents the Italian program of reforms and investments for the achievement of a series of objectives among which emerges the one related to ecological transition. According to the forecasts contained in the PNRR, in order to achieve the objectives of decarbonization of the mobility sector, a circulating fleet of about 6 millions electric cars is estimated by 2030 for which about 31.500 public fast charging points are needed [6]. Assuming that it is possible to actually reach the pre-established target of public charging stations, what is interesting for those who manage the electricity distribution network is to know how these will be distributed along the national territory. The contribution of such a quantity of charging points generally involves an increase in the load on the distribution network. All of such factors are therefore strongly linked to the spread of electric vehicles. It is reasonable to hypothesize that the concentration of charging points in the coming years will be higher in those areas where there will be a high percentage of electric cars with respect to the whole circulating car fleet of that area. This is the reason why to plan the interventions on the distribution network, it is necessary to be able to estimate the diffusion of electric vehicles. To the best of our knowledge no pre-existing database is present for the italian territory designing the main important features for electric cars spread.

For this purpose, we initially identify from the existing literature some of the main factors influencing the diffusion of electric mobility:

- *The economic incentives*: the Italian “*Ecobonus*” is the measure promoted by the Ministry of Economic Development that offers grants for the purchase of low-emissions vehicles. This incentive was introduced in 2019 with a funding of 60 millions of euros and was then renewed for both 2020 and 2021 with much higher funding of 262 and 283 million respectively [7]. This represents a fundamental driver for the spread of electric mobility, especially today, when the average price still exceeds that of conventional internal combustion vehicles. Based on a statistical report published by ANFIA (National Association of the Automotive Industrial Chain), with the introduction of the “Ecobonus” the share of electric cars sold to individuals rose from 17.5% in 2018 to 26% in 2019 (up to 33% in the first half of 2020) [8].
- *The recharging infrastructure*: as already mentioned above, the charging structure for electric cars is the dominant factor, especially considering large cities, where the availability of private parking spaces or garages is more limited, and the capillarity of public charging points becomes decisive for the spread of purely electric vehicles. The network of charging infrastructure for electric vehicles in Italy follows growth trends that are comparable with those of electric cars. Despite the continuous growth, however, it is necessary to highlight the need for a widespread network of high-power recharges, the so-called fast and ultra-fast (from 50 kW upwards), which allow a recharge to 80% even in less than 10 minutes. Currently, more than 70% of public recharges have a power of around 22 kW ("normal charge"), while high-power recharges will have to be quickly installed on the main routes and in urban areas paying particular attention to the choice of installation locations (e.g. railway stations, fuel distributors, etc.) [9].
- *The price*: Italian citizens still perceive the limit on the purchase price as binding. If on the one hand this is linked to the low propensity of people to evaluate the Total Cost of Ownership (TCO) of the goods they buy, on the other hand there is no doubt that the purchase prices still do not allow, despite the incentives, access to this type of solution to citizens belonging to the lowest income brackets. In particular, the battery pack weighs 40% on the cost of the vehicle, but the birth of new production poles, especially in Europe, is expected to bring the price below the threshold of 100 $/kWh within 2025, identified by several authoritative subjects as the target that allows to electric cars’ price to be competitive with the one of ICE vehicles.

Considering all of the aforementioned factors, in this paper we present an analysis of electric mobility, through which the main factors influencing its diffusion have been identified. In this way we could tackle the research question of estimating the distribution of the fleet of electric cars circulating on the Italian national territory, identifying the possible features that may have a fundamental role in their diffusion. This objective was pursued by applying machine learning techniques. Machine learning, in fact, has been proved to be nowadays successful in a wide variety of different fields [10–21] from bioinformatics to computer vision, from natural language processing to recommender systems. To the best of our knowledge, however, data mining and machine learning techniques have never been applied to the study of EVs and prediction of circulating number of vehicles, especially referring to the Italian scenario case. This is probably also due to the lack of public datasets available for the study. In our case, therefore, we collected a novel database from public repositories for the purpose of this study using Italian databanks as for instance of ISTAT and Terna [22, 23] (we will describe the data crawling process in details in Section 2).

To study the phenomenon in depth, the research was deployed in such a way that we could perform modelling at different scales to give a general Italian overview of the scenario. A first analysis was carried out on an Italian regional scale, which was then followed by an Italian provincial one. In both scenarios we compared four supervised machine learning approaches. The performances showed to be quite comparable among the machine learning approaches implemented, with a slighter better performance obtained through the use of the Extreme Gradient Boosting Regressor (XGBR).

Both from the national and the regional case, it emerged that among the features that played a more central role stand out those that are directly connected to the main drivers that influence the spread of electric mobility, such as the price and the presence of charging points.

Subsequently we further refined the research, through the creation of a novel dataset obtained from a survey distributed in a sample of the population. These data were obtained through the creation and diffusion of a survey. Also in this case, the same machine learning models used in previous analyses were applied. The results obtained further confirmed the hypotheses made regarding the main factors of diffusion of electric mobility, thus allowing to make a direct comparison on the three different scales considered for the analysis.

The paper presents several novel contributions. To the best of our knowledge this is the first time that a machine learning approach is used for the purpose of studying electric cars in the Italian scenario. Moreover the dataset used in the study is novel, and collected with the purpose of further investigating the possible factors affecting the electric cars Italian mobility. Furthermore the survey proposed is newly designed, and it allowed to have a three dimensional analysis of the electric cars scenario in Italy, which is, to the best of our knowledge, the first time this is done in the literature.

The paper is organized as follows. In Section 2 we present a comprehensive overview of the dataset collected, showing the public repository we used to collect it, as well as a description of the survey modelling. In Section 3 we describe the machine learning approaches used in the study, while in Section 4 we thoroughly describe the experimental setting designed. In Section 5 we draw conclusions and future perspective of the study proposed.

## 2. Dataset

The several features that constitute the datasets were crawled separately and downloaded from online available databases, mainly from ISTAT [22] which represents the Italian National Institute of Statistics. Subsequently, data were aggregated and merged to obtain a single dataset. We organized the data crawling phase in order to get two types of datasets: one that would describe the Italian scenario on a regional scale and a second one which would describe the same scenario but on a provincial scale. In this way, in fact, it would have been possible to compare the defined models, highlighting in particular which of the features had a more decisive role in the prediction. In addition to the regional and provincial analysis, a third analysis was carried out on a dataset made from the data collected through an original survey that we designed. This allowed us to collect completely novel data on which to apply the same predictive models used in the two previous cases, also allowing us to make additional considerations on the factors highlighted for the regional and provincial analysis.

We released the data and the code for our project at: https://github.com/GiovannaMariaDimitri/A-machine-learning-approach-to-analyse-and-predict-the-electric-cars-scenario-the-Italian-case

- **Regional and provincial datasets**

The construction of regional and provincial datasets took place in three steps:

1. ***Variables identification***: among the identified factors which may influence the diffusion of electric mobility, the most commonly reported ones were the presence of charging infrastructure, the price of electric cars and consumers’ sensitivity towards a more sustainable mobility. Therefore, the research focused on finding all of those variables that could be identified with the factors or that could somehow be related to them. In addition to those already mentioned, we identified further elements such as the growth of renewable energy sources, more precisely photovoltaic panels, and the presence of increasingly electric company fleets. Although sometimes they are not treated by the existing bibliography, photovoltaic panels could contribute to the growth of electric cars since they would allow consumers to recharge batteries at no-cost by using the energy produced by the panels.
2. ***Data gathering***: once the variables to use for the composition of the datasets were identified, it was necessary to search and download the data relating to each of them from online data sources. Initially, given the higher availability, the data were downloaded on a regional scale, and only later the field of research was restricted also at the provincial level. These were mainly collected from the database of the National Institute of Statistics, better known as ISTAT [24, 25].
3. ***Data aggregation***: finally, once the data for all the variables had been downloaded, they were aggregated to obtain a single dataset. Obviously, parallel to the aggregation phase, a series of data transformations and corrections were carried out, mainly due to the fact that these were available in different formats. These procedures, as well as all the subsequent analysis that led to the creation of predictive models, was carried out entirely using the Google Colaboratory service (usually abbreviated as "*Colab*" [26]).

The regional and provincial variables are summarized in Table 1.

**Table 1:** Describing features types, divided for category which have been collected in our data collection process.

CATEGORY	FEATURE	TYPE	REGION	PROVINCE
Demographic	Male	Float (%)	✔	✔
	Female	Float (%)	✔	✔
	Residents	Int	✔	✔
	Max25	Float (%)	✔	✔
	Max50	Float (%)	✔	✔
	Max75	Float (%)	✔	✔
	Max100	Float (%)	✔	✔
Economic	GDP Per Capita	Float	✔	✖
	Micro enterprise	Float (%)	✔	✔
	Small enterprise	Float (%)	✔	✔
	Medium enterprise	Float (%)	✔	✔
	Large companies	Float (%)	✔	✔
	Total enterprises	Int	✔	✔
Circulating Cars Fleet	Total cars	Int	✔	✔
	Gasoline	Float (%)	✔	✔
	Diesel	Float (%)	✔	✔
	Biogas	Float (%)	✔	✔
	Electric	Float (%)	✔	✔
	Euro 3	Float (%)	✔	✔
	Euro 4	Float (%)	✔	✔
	Euro 5	Float (%)	✔	✔
	Euro 6	Float (%)	✔	✔
Sustainability	Potential Pollution	Float	✔	✔
	PM10 exceedances	Int	✔	✔
Electric	Industrial consumption	Float (%)	✔	✖
	Agricultural consumption	Float (%)	✔	✖
	Tertiary consumption	Float (%)	✔	✖
	Household consumption	Float (%)	✔	✖
	Total consumption	Float	✔	✔
	Nr. Photovoltaic panels	Int	✔	✔
	Installed power	Float	✔	✔
	Net production	Float	✔	✔
Charging Infrastructure	Nr. Recharging points	Int	✔	✔
	Recharging points density	Float	✖	✔

Table describing features types, divided for category which have been collected in our data collection process. In columns 4 and 5 we describe which ones were present for the regional or for the provincial case respectively. Max25, Max50, Max75, Max100 are features indicating the demographic subdivision by age. Euro 3, Euro 4, Euro 5, Euro 6 indicates the European emission standards for passengers cars and they are defined on the year of car’s registration: 2000, 2005, 2009 and 2014 respectively.

The data presented are divided into macro category, and for each macro category we report the name of the feature, the type of feature (if floating point or integer). A green tick highlights if the relevant feature is present for the regional or provincial case or both.

The first column of Table 1 represents the category; the second column the various features available for the particular category; the third column represents the type of feature (float or integers) and the last two columns indicate if the feature is present for the regional or for the provincial dataset. For example the feature Residents is of type INT as it is representing the number of citizens, and is present both for the regional and provincial dataset. Instead, as a further example, for the category Circulating Car Fleets we have the feature Gasoline, Biogas or Electric which are float, as they indicate the percentages of cars of that type which are available in the datasets. In both cases, in the example, we have them available for the regional and the provincial datasets.

- **Survey dataset**

In this subsection we describe the process that led us to the creation of the dataset obtained from the survey we designed and distributed. The process can be discretized in three steps.

1. ***Survey creation***: similarly, to what was done in the research phase for the creation of regional and provincial datasets, also in this case we started from the main factors that play a fundamental role in the spread of electric mobility. However differently from before, the initial objective was not to search for the variables connected to the main factors, but to create the questions to include in the survey which are related to them. Moreover, differently from the previous case, we could also add further questions to compare the features identified as important at the previous step with respect to the previous case in which only online available data could be used. In particular, for example, we added questions investigating aspects such as economic incentives and fuel economy, both of which had not been deepened in previous analysis due to the unavailability of data. In conclusion, a total of 44 questions were formulated and circulated through the obtained survey. In Table 2 we present an overview of the questions made. For the sake of description questions have been grouped based on the object of investigation such as users’ habits with the car, statistical information on the cars owned by the respondents and the purchase propensity. For what concerns survey ethical approval please see the details in the Methods section. In S1 File, S.1 Section we present the complete questionnaire created.
2. ***Survey administration***: in order to reach as many respondents as possible, the method of administration chosen for the diffusion of the survey was the online one. More precisely, the used tool is Google Forms, which is part of the free and web-based Google Docs Editor suite offered by Google [27]. The survey was left open for approximately one month, more precisely from 9 August (opening date) until 12 September 2021 (closing date). During this time period it was possible to collect the answers of a total of 300 users, coming from different Italian regions and provinces.
3. ***Dataset creation***: once the response collection period was over, the relevant data was downloaded in CSV (comma-separated values) format directly from Google Forms. Subsequently, the data were uploaded to Google Colaboratory, as was also the case with regional and provincial analyses.

**Table 2:** Describing features types, divided for category which have been collected through the distribution of our survey.

FEATURES	QUESTIONS
Screening questions	1,2
Cars fleet questions	3 to 12
Habits with the car	13 to 17
Habits of electric cars owners	18 to 22
Electric mobility factors	23 to 28
Purchase propensity	29 to 31
Place of residence	32 to 35
Photovoltaic panels	36 to 40
Personal questions	41 to 44

*Table describing features types, divided for category which have been collected through the distribution of our survey. In column 1 we describe the features and in column 2 the relative questions in which the features were investigated. A detailed description of all of the questions is reported in S1 File.*

## 3. Methods

One of the main objectives of this study is to compare the analyses carried out on different Italian scales in order to highlight the similarities and differences and paying particular attention to which were the features most significant in the various machine learning models. Consultation for what concerns the ethical approval was requested to the CAREUS ethics committee of the University of Siena (https://en.unisi.it/research/ethics-committee-research-human-and-social-sciences-careus). The committee stated that given the questions of which the survey was composed of, which do not imply the presence of sensible and reserved information, the CAREUS did not need to express any ethical approval concerning the research hereby presented. This is also reported in the ethical statement section of the paper. Informed consent was obtained verbally by the distributor of the survey and a privacy statement included in the beginning of the survey. No minors were included in the survey study.

In order to be able to compare the results obtained, we applied a suite of machine learning models to the three datasets obtained. Since the purpose of the paper was not to propose a new machine learning model, but to test machine learning in a new and uncovered field of research, we decided to use 4 well known and established supervised machine learning algorithms: *Linear Regression*, *Ridge Regression*, *Decision Tree Regressor*, *Extreme Gradient Boosting Regressor*.

We will now give a brief overview of the various models, before describing the results obtained in section 4.

- ***Linear Regression***: this well known and widely used model assumes a linear relationship between the input variables (*x*), also known as the *predictors*, and the single output variable (*y*), which is also called the *response* variable. Therefore, a linear regression aims at finding a relationship among one or more predictors, which are used to compute the value of the response variable. More specifically, the output variable (*y*) can be computed from a linear combination of the input variables (*x*) [28]. For this study, we refer to Multiple Linear Regression, that is the case in which the number of predictors is greater than one. In this case, the expression is the one represented by Eq 1:

$$y=\beta_{0}+\beta_{1}x+\cdots +\beta_{m}x+\varepsilon$$ (1)

where *y* is the dependent variable, *x*_*i*_ is the i-th independent variables, *m* is the number of predictors, *β*_*i*_ the polynomial coefficients of *x*_*i*_ and *ε* the residual. Assuming ***X*** as the predictors matrix and ***Y***, ***β***, ***ε*** as the vector of the dependent variable, the coefficient vector and the residuals vector, the standard least-square error method can be used to compute the coefficient vector *β*, and the formula is presented in Eq 2:

$$\beta ={\left(X^{T}X\right)}^{-1}X^{T}Y$$ (2)

Once the regression coefficients have been computed, Eq 1 can be used to predict the values of the dependent variable (*y*) related to new input values (*x*) [29].

- ***Ridge Regression***: it is one of the best alternatives to the standard linear regression method in the case of multicollinearity. The phenomenon of multicollinearity occurs whenever two or more predictors in a regression model are moderately or highly correlated. In this case, the explanatory variables are dependent on each other. The main consequence is the fact that small changes in the input data can highly affect the estimates of regression coefficients and this can be translated into a model’s loss of robustness [30]. The ridge regression algorithm aims at solving the problem of multicollinearity by adding a regularization term when estimating the regression coefficients. Consequently, Eq 3 can be rewritten as follow:

$$\beta ={\left(X^{T}X+\lambda I\right)}^{-1}X^{T}Y)$$ (3)

The purpose of the parameter *λ* is to reduce the values of the estimated coefficients. In this way, all variables that do not play an important role in estimation, have coefficients close to zero, but never completely zero [31].

- ***Decision Tree Regression***: in decision tree modelling, an empirical tree represents a segmentation of the data that is created by applying a series of simple rules [27]. The tree is composed by a root node, which contains all data, a set of internal nodes (splits) and a set of terminal nodes (leaves).

Since in the case of our study, the target variables of the different analyses are continuous, therefore the algorithm is a decision tree regressor. The construction of a regression tree is based on binary recursive partitioning, which is an iterative process that splits the data into partitions. More precisely, starting from the root node, the algorithm breaks the data using every possible binary split and then the best partition is selected. The quality of a partition is measured using the reduction of variance, therefore the best split is the one that minimizes the variance [32]. The splitting process is applied to each node of the tree and it continues until each of them reach a minimum pre-defined size of observations and becomes a leaf node.

- ***Extreme Gradient Boosting Regressor*:** it is a specific application of the Gradient Boosting method, which uses more accurate approximation to find the best tree model. More precisely, the Gradient Boosting method produce a predictive model in the form of an ensemble of weak prediction models, typically decision trees [33]. Generally, Gradient Boosting aims to predict model errors. In particular, model prediction errors are computed as the difference between the real and predicted values and then Gradient Boosting usually uses a regression tree to estimate new residuals. This procedure is repeated iteratively, and the residues are progressively reduced, improving the accuracy of the model [34].

## 4. Experiments and results

### 4.1 Italian regional analysis

The regional dataset consists of 33 features, which represent the variables that have been obtained by downloading the data from the ISTAT databases as described in Section 2. Depending on the availability of the data, it was possible to construct a total of five datasets, one corresponding to each year from 2015 to 2019 (including both extreme years). As we will see, not all features will be used to define the predictive models. In fact, only half of them, more precisely 15, will be useful for estimating the percentage of electric cars in the region.

**Fig 1:**
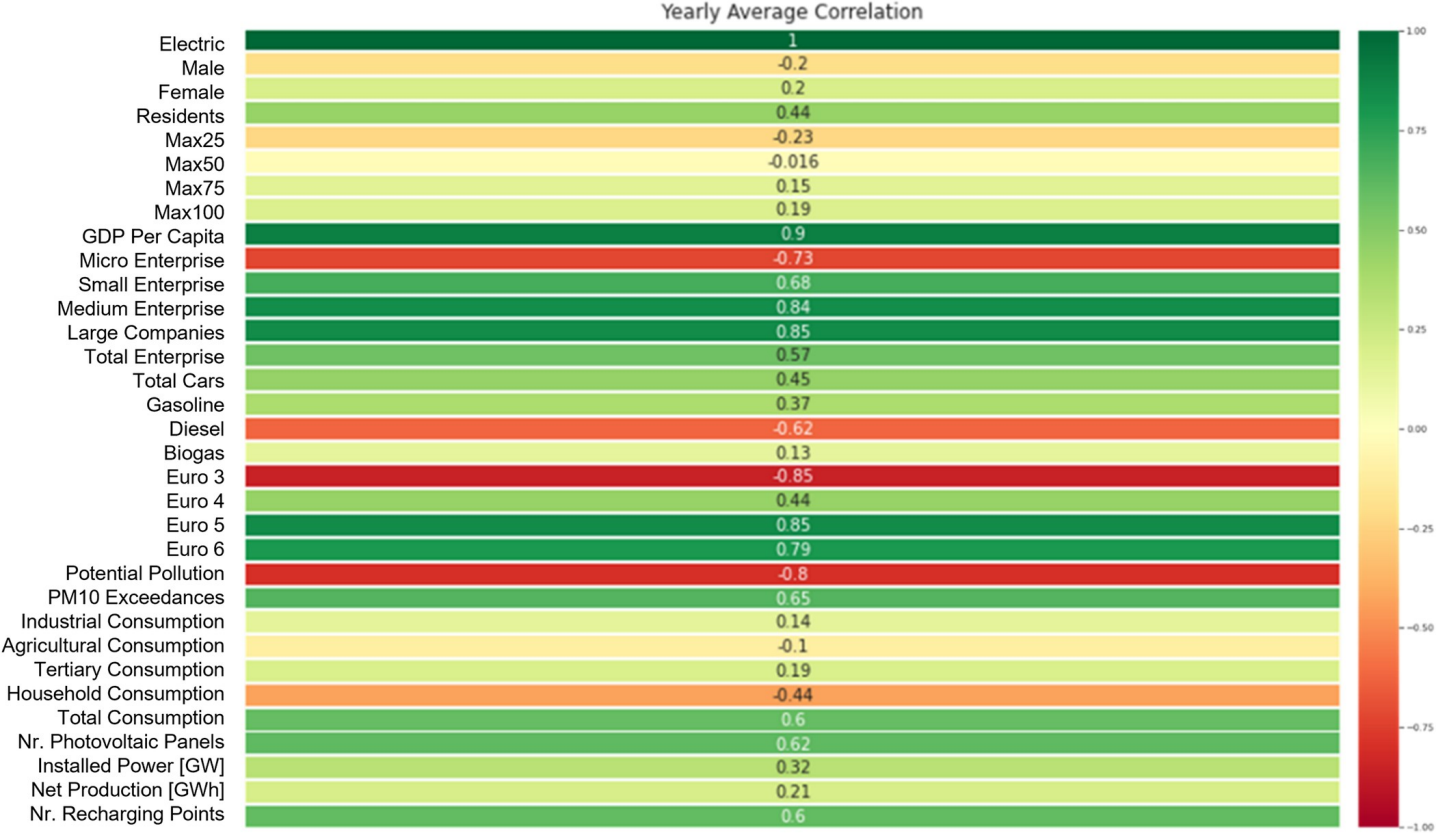
Regional features average Pearson correlation coefficient with the target variable (regional percentage of EVs). The average is computed as the mean value of yearly correlations. Values are included in the range [–1,1].

To perform a features selection step, in order to identify the variables that are most important for the definition of the models, the correlation matrices among features collected in our novel regional dataset, were computed. For each of the features we computed the average of the correlations of the five years considered. In this way we took into account a unique correlation value with the target variable. The reason for this choice is due to the fact that in this way we could consider a single set of features valid for each of the five datasets. Fig 1 shows the average value of the features’ correlations computed as the mean value of those related to the year from 2015 up to 2019.

The selection of the variables considered most relevant for the creation of the predictive models has been made through the definition of a minimum absolute correlation’s threshold equal to 0,5. The choice of this threshold was made in an iterative way. Initially, the models were defined using all the features of the dataset, thus placing the threshold equal to zero. Subsequently, the value of the correlation threshold was increased in such a way as to exclude part of the variables from the model and the performance was compared with the previous models. This procedure was repeated iteratively until the value of 0.5 was reached, which maximizes the performance of the model while avoiding an excessive reduction of the dimensionality of the dataset.

Some comments concerning the results obtained in this pre-processing step. It is not surprising that among the variables that have absolute value correlations equal or higher than 0,5, it is possible to detect those that are closely related to the main factors that influence the spread of electric cars. Among these, the noteworthy ones are:

- ***GDP Per Capita (PIL)*:** which can be associated with the price factor, assuming that in the richer regions the propensity to spend more on the purchase of electric cars is higher.
- ***Polluting potential* and *PM10 exceedances***: which can be associated with the sustainability factor. In fact, the percentage of electric cars is higher in regions where the polluting potential is lower and where there has been a higher number of times when the limit threshold of PM10 particulate has been exceeded. Consequently, it can be assumed that policies are being adopted in these regions in order to raise the awareness of air quality safeguards and to accelerate the adoption of electric cars.
- ***Number of charging points*:** which is the main factor in the spread of electric cars. It is therefore not surprising that there is a direct proportionality between the number of charging points and that of electric cars.
- ***Number of photovoltaic panels*:** which, although it is not frequently mentioned in the bibliography related to electric mobility, is actually a potentially relevant factor in favor of an increase in circulating electric cars. In fact, in addition to the thematic of sustainability, it can also be linked to the maintenance cost of an electric vehicle. If a potential user has a private charging station, he/she could recharge the battery of his car at no cost, thanks to the energy produced by the photovoltaic panel. Obviously, this is a clear element in favor of the adoption of an electric vehicle.

**Fig 2:**
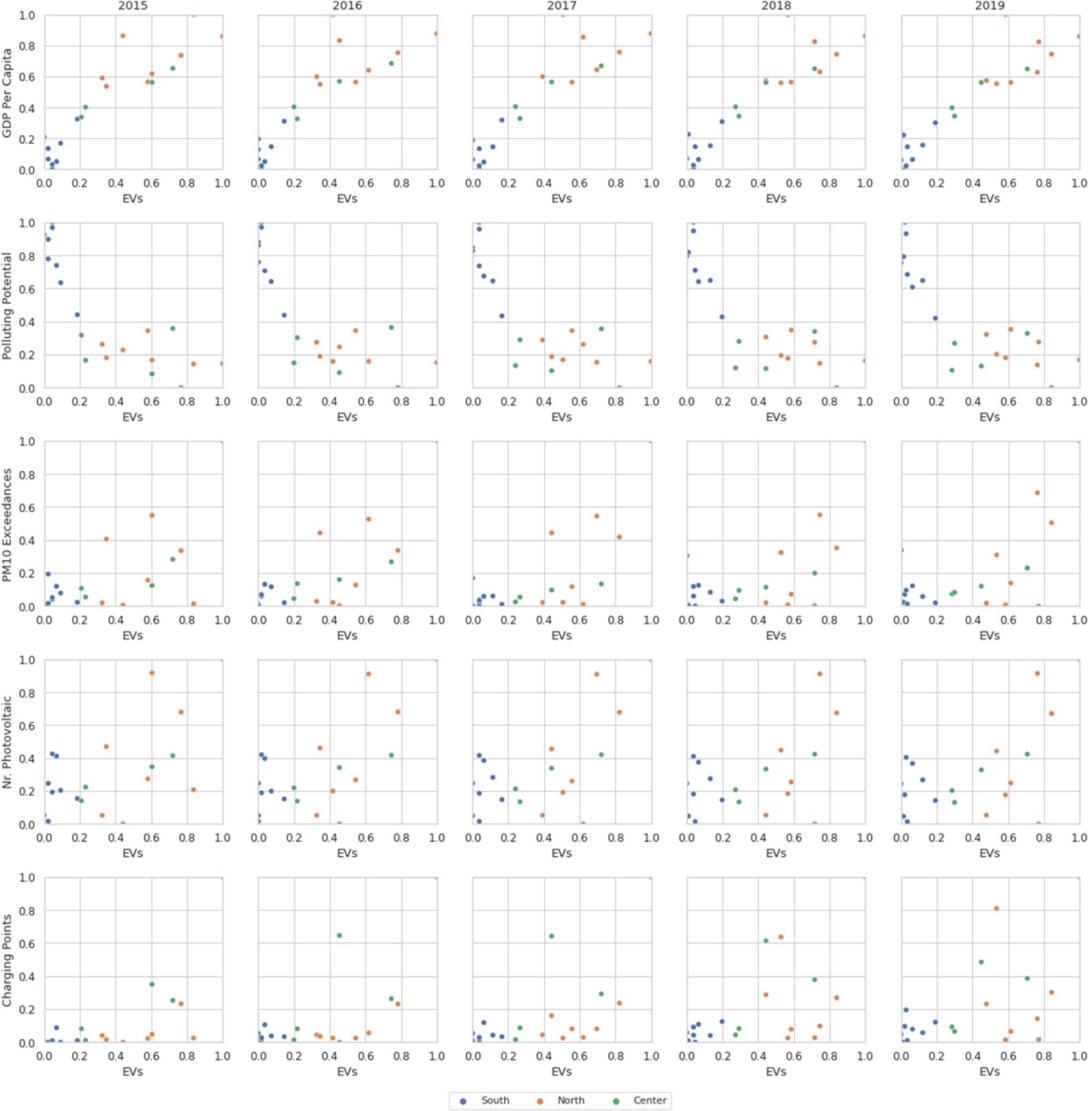
Scatter plot matrix with the target variable (regional percentage of EVs) of the following features: *GDP Per Capita (PIL)*, *Polluting Potential*, *PM10 Exceedances*, *Number of Photovoltaic Panels* and *Number of Charging Points*.

All the considerations made so far are also confirmed in the scatter plots shown in Fig 2, which relate the just mentioned variables with the target variable (the regional percentage of EVs) and highlight the differences among the North, Center and South of Italy on annual basis.

Each of the machine learning models presented in section 4 was applied for each yearly collected dataset. Performances were then evaluated comparing predicted and real values, for the subsequent year.

**Fig 3:**
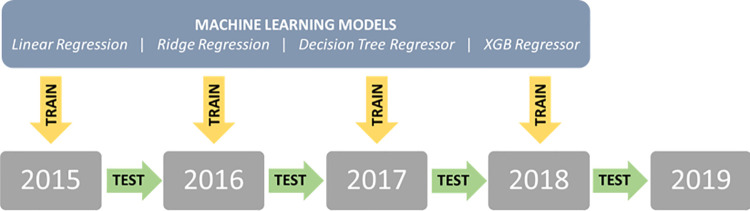
Workflow of the Training and testing procedure of the model for the regional and provincial analyses.

Fig 3 graphically illustrates the followed procedure.

The metrics we used to evaluate individual models (typically used in the case of regression) were:

- ***Root Mean Squared Error (RMSE)*:** it represents the square root of the second sample moment of the differences between predicted values and observed values or the quadratic mean of these differences. Assuming that $\hat{y}$ is the predicted value and *y* the original value, for *n* samples, the formula is the one shown by Eq S1 in S.3 Section of S1 File.
- ***The coefficient of determination* (*R***^**2**^**):** it is an index that measures the link between the variability of the data and the correctness of the statistical model used. The most generic formulation is shown in Eq S2 in S.3 Section of S1 File.

Using the just described metrics, it was possible to evaluate and compare the four different regression models applied to the various datasets.

Table 3 summarizes the main results. The rows correspond to the datasets, while the columns are the regression models for each of which the two metrics mentioned above are shown.

**Table 3:** Performance for the four different applied models in regional analysis: Linear Regression (LR), Ridge Regression (RR), Decision Tree Regressor (DT), Extreme Gradient Boosting Regressor (XGBR). The last row shows the metric’s average value for the four years.

	LR		RR		DT		XGBR	
	RMSE	R^2^	RMSE	R^2^	RMSE	R^2^	RMSE	R^2^
2015	0.11	0.86	0.12	0.82	0.10	0.87	0.10	0.87
2016	0.07	0.94	0.11	0.87	0.06	0.96	0.07	0.95
2017	0.10	0.90	0.11	0.88	0.05	0.97	0.05	0.97
2018	0.20	0.58	0.06	0.97	0.08	0.94	0.03	0.99
Average	0.12	0.82	0.10	0.89	0.07	0.94	**0.06**	**0.95**

Looking at the values shown in Table 3, it is possible to see how the performance of the trained models on the 2015 dataset is on average lower than the others. This is probably due to the fact that electric cars have had a more marked growth in the following years and the data dating back to 2015, although present, were not sufficient to be able to provide an accurate estimate of the trend of the following years. For what concerns the comparison between the different applied models, observing the last row of the table which shows the average calculated on the four datasets, it turns out that the most performing is the *Extreme Gradient Boosting Regressor (XGB)*, albeit slightly if compared to the *Decision Tree Regressor (DT)*. In fact, by looking at the values of the metrics, we can see that the coefficient of determination and the RMSE are both 0.1 higher and lower in the case of XGBR.

**Fig 4:**
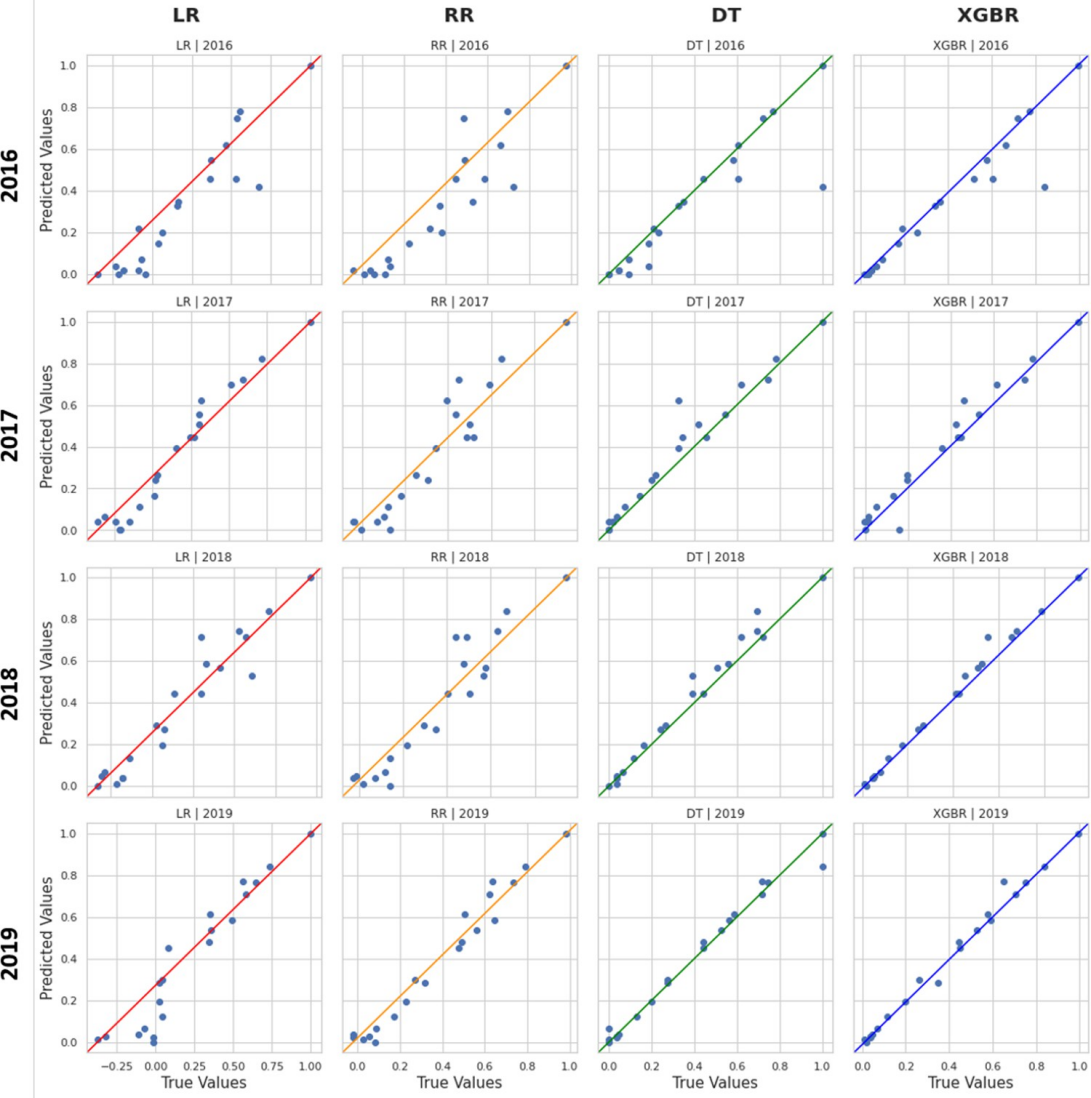
Scatter plots matrix for regional models’ performance. For each scatter plot, the true values are reported on the x-axis, while the predicted ones are reported on the y-axis. The rows of the matrix represent the years, while the columns represent the implemented model: Linear Regression (LR), Ridge Regression (RR), Decision Tree Regressor (DT), Extreme Gradient Boosting Regressor (XGBR).

Fig 4 shows the matrix of scatter plots that compares the predicted values with the real ones and it graphically summarizes what we described so far. Rows represent datasets, and columns show applied models.

### 4.2 Italian provincial analysis

We repeated the experiments described so far for the analysis of the Italian provinces. Also in this case the main objective was to identify which are the variables that have a greater relevance for the purposes of the model. We first obtained the correlation matrix to highlight the value of the correlation that the individual features have with the target variable for each dataset (for each year).

**Fig 5:**
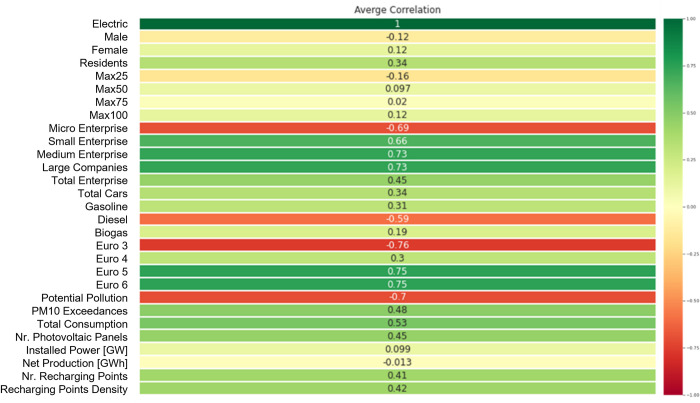
Provincial features average correlation with the target variable (provincial percentage of EVs). The average is computed as the mean value of yearly correlations. Values are included in the range [–1,1].

Fig 5 highlights the average value of the features’ correlations computed as the mean value of those related to the year from 2015 up to 2019.

It is possible to notice that both at regional and provincial level, the variables indicating the percentage of companies present in the territory, divided by size, are highly correlated with the target variable.

The reason why these variables have been included in the construction of the various datasets, must be identified in the fact that they can be associated with the company’s car fleets. Given the absence of company fleet data on online databases, we hypothesized that a greater territorial concentration of large companies means that these companies are more likely to have company fleets that include electric cars. Analyzing the correlation values, this assumption seems to be confirmed. In fact, it is noted that where the concentration of very small companies is greater, the percentage of electric cars is lower. We can further notice this, observing the trend highlighted in Fig 5, where it is possible to notice in the opposite proportionality that micro enterprises and large companies have with the variable target. Among the other features that appear to have significant correlations we find again the polluting potential, the PM10 exceeding times, the number of photovoltaic panels and charging points.

**Fig 6:**
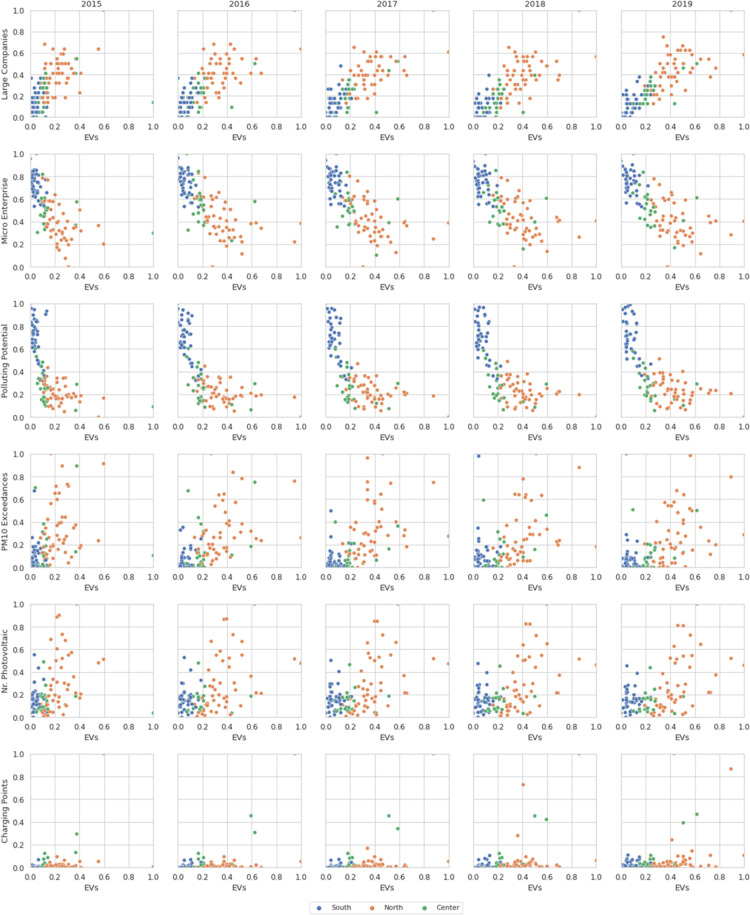
Scatter plots matrix with the target variable (regional percentage of EVs) of the following features: *Large Companies*, *Micro Enterprises*, *Polluting Potential*, *PM10 Exceedances*, *Number of Photovoltaic Panels* and *Number of Charging Points*.

Fig 6 shows the bivariate visualization of all these features with the target variable, that is the percentage of electric cars circulating in the province.

**Fig 7:**
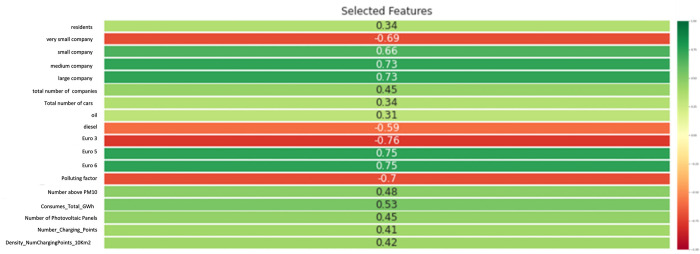
Regional selected features with correlation equal or higher than 0.3 in absolute value. Values are included in the range [–1,1].

By iteratively evaluating the performance of the forecasting models as the minimum correlation threshold for variables changes, a value equal 0,3 to which performance is optimized has been reached. Therefore, the selected features for the definition of the models are shown in Fig 7.

We subsequently applied the regressive models, similarly to what has been seen for the analysis on a regional scale and using the same types of metrics for the evaluation. The results obtained are as shown in Table 4.

**Table 4:** Performance for the four different applied models in provincial analysis: Linear Regression (LR), Ridge Regression (RR), Decision Tree Regressor (DT), Extreme Gradient Boosting Regressor (XGBR). The last row shows the metric’s average value for the four years.

	LR		RR		DT		XGBR	
	RMSE	R^2^	RMSE	R^2^	RMSE	R^2^	RMSE	R^2^
2015	0.13	0.52	0.15	0.41	0.17	0.21	0.12	0.63
2016	0.08	0.81	0.09	0.79	0.07	0.88	0.04	0.96
2017	0.08	0.83	0.09	0.78	0.08	0.82	0.04	0.96
2018	0.08	0.86	0.08	0.86	0.07	0.88	0.04	0.96
Average	0.09	0.76	0.10	0.71	0.10	0.70	**0.06**	**0.88**

**Fig 8:**
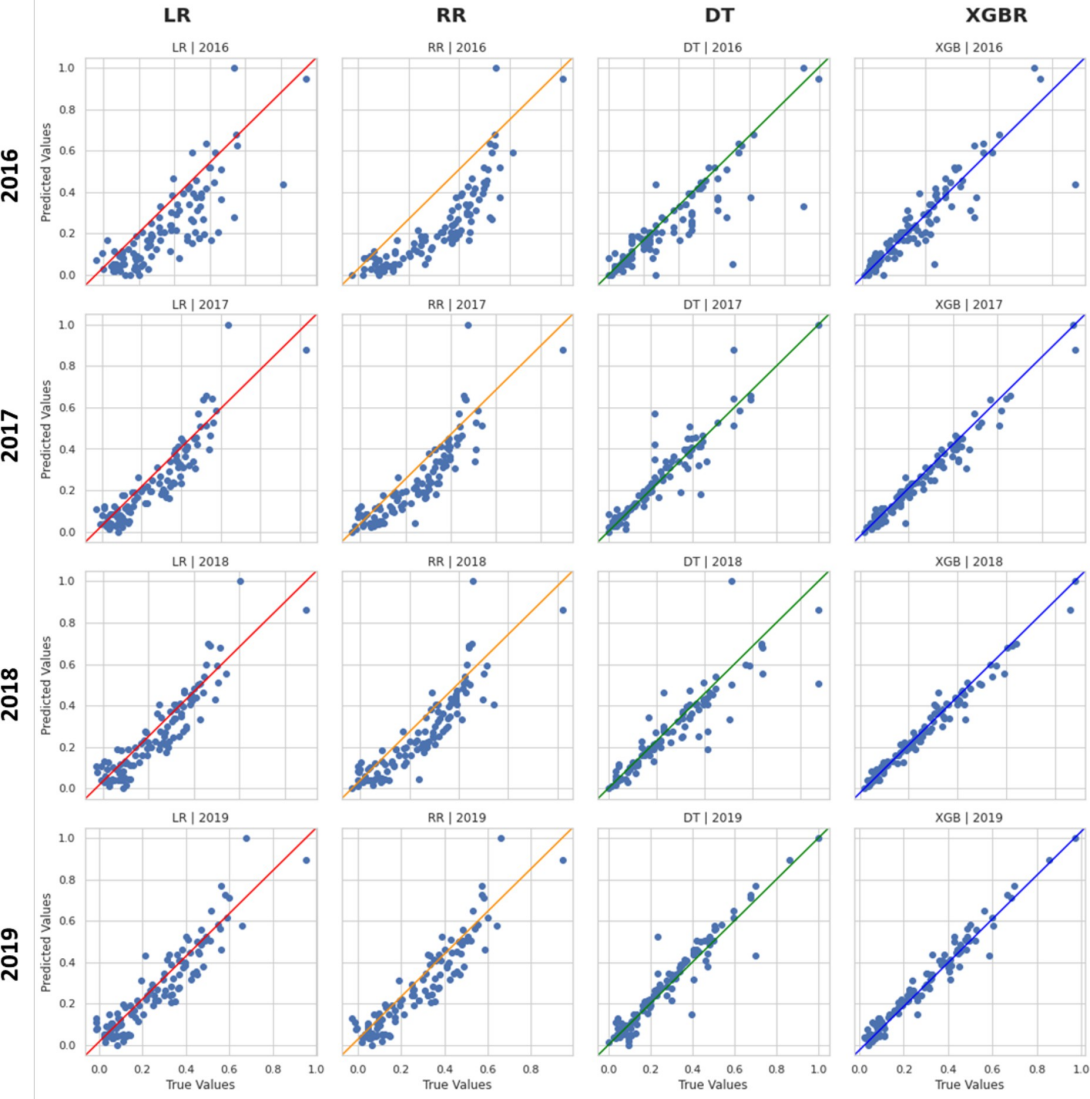
Scatter plot matrix for provincial models’ performance.

Looking at the values, even in this case, the performance of the 2015 dataset is significantly lower if compared with those of the other datasets. Performances are quite comparable among the 4 models proposed. However, among them, the XGBR model turns out to be the best predictive model, for which the coefficient of determination is maximized and the RMSE is minimized. More precisely, the *R*^2^ score is 0.88 and the RMSE is 0.06. In conclusion, the graphical visualization of the models’ performance is shown in Fig 8.

### 4.3 Survey analysis

As a further step of our analysis, we decided to conduct a survey to go down in the analysis hierarchy of our study. Starting from the regions, in fact, we then zoomed into the provinces, to eventually conduct a survey that considered a sample of the Italian population. The number of respondents to the survey was 300, each of whom answered the questions that compose the survey. The final survey dataset is therefore composed of 300 rows, each of which corresponds to a user and 44 features, which represent the information collected through the survey questions. In the rest of this section we will describe the main results obtained. In S1 Section in S1 File we report the integral survey questionnaire and structure. Moreover in S2 Section in S1 File we report preliminary data visualization and descriptive statistical analysis of the survey questionnaire answers.

**Fig 9:**
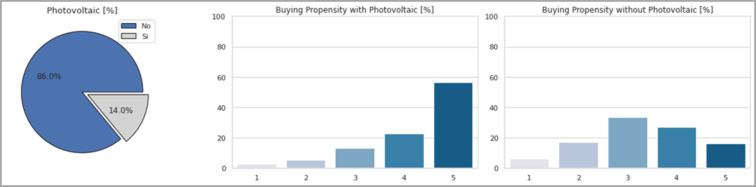
Respondents’ opinion towards photovoltaic panels. Starting from the left, it shows how many respondents own at least a *Photovoltaic Panel*, their *Buying Propension with* and *without it*.

For what concerns the answers related to the factors that may or may not favor the adoption of an electric vehicle, Fig 9 shows the results related to the presence of photovoltaic panels.

The presence of photovoltaic panels, as already stated above, could in fact represent an element in favor of the purchase of an electric car, since it would allow the user to recharge the battery of his car using the energy produced in a sustainable way, therefore at no cost. Regarding this concept, we noticed how only 14% of respondents currently own photovoltaic panels. Despite this, the role that this aspect has in relation to electric mobility is evident. In fact, thanks to the presence of photovoltaics, the propensity to purchase goes from 43% (in the absence of photovoltaics) to almost 80% (with possession of photovoltaics). These values were obtained by adding the percentages for replies to questions "4" and "5".

**Fig 10:**
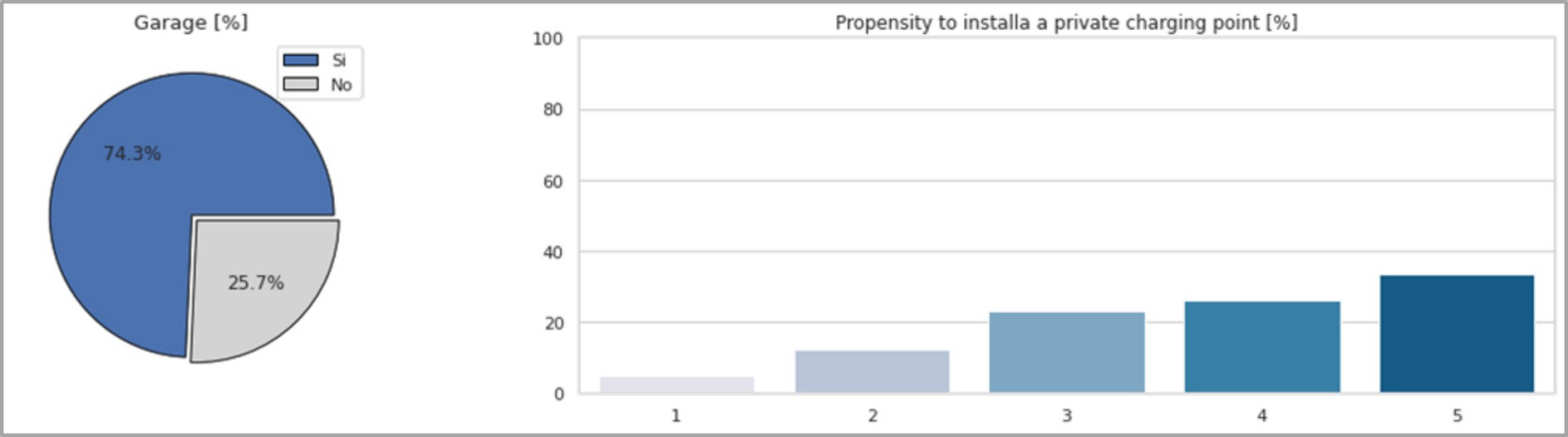
The percentage of users owning a *Garage* and their propensity to *Install a private charging point*.

Always connected to battery charging, almost three-quarters of respondents have private parking for their car and assuming they own an electric car more than half (58.6%) would be inclined to invest in the installation of a private charging station. These percentages are illustrated in Fig 10.

**Fig 11:**
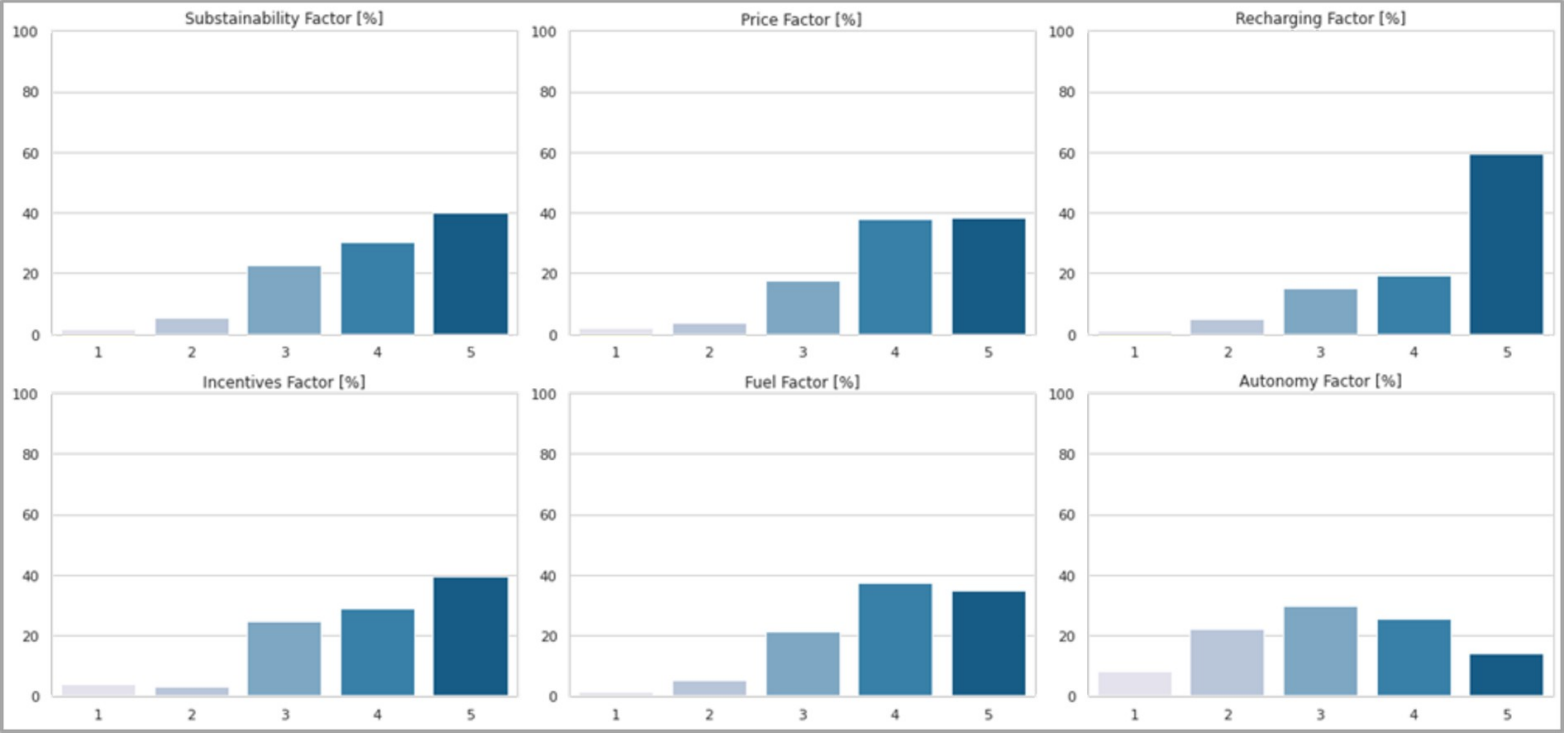
Respondents’ opinion towards the main factors influencing the spread of electric cars. They had to assign a weight from 1 to 5 to each of the factors.

One of the main objectives of the survey is to investigate the weight that the main factors that can influence the spread of electric cars have. In this regard, users were asked to quantify the influence they have on them. As can be seen from Fig 11, all the factors are highly influencing for users, since for each of them about 70% of users answered "4" or "5", thus indicating a considerable weight of that factor. From a more detailed point of view, if on the one hand sustainability, the presence of incentives and fuel economy are around 70%, on the other hand the price is considered a determining element by 76.3% of users and the problem of the availability of charging points reaches even 80%. The latter obtained the highest score from about 60% of respondents, confirming itself as the main driver for the spread of electric cars. The only exception to this is the autonomy of the batteries. Although it is often cited in the bibliography as one of the main obstacles of electric mobility, in this case the respondents had equally opposed opinions. From the graph it is possible to see the symmetry of the results, with about 30% providing an impartial answer. This factor turns out to be more subjective if compared with others, since it is directly related to the user’s driving habits.

Before moving on to the machine learning and modelling description of the analysis, the main statistical data relating to those who responded to owning at least one electric car are reported in Fig 11.

**Fig 12:**
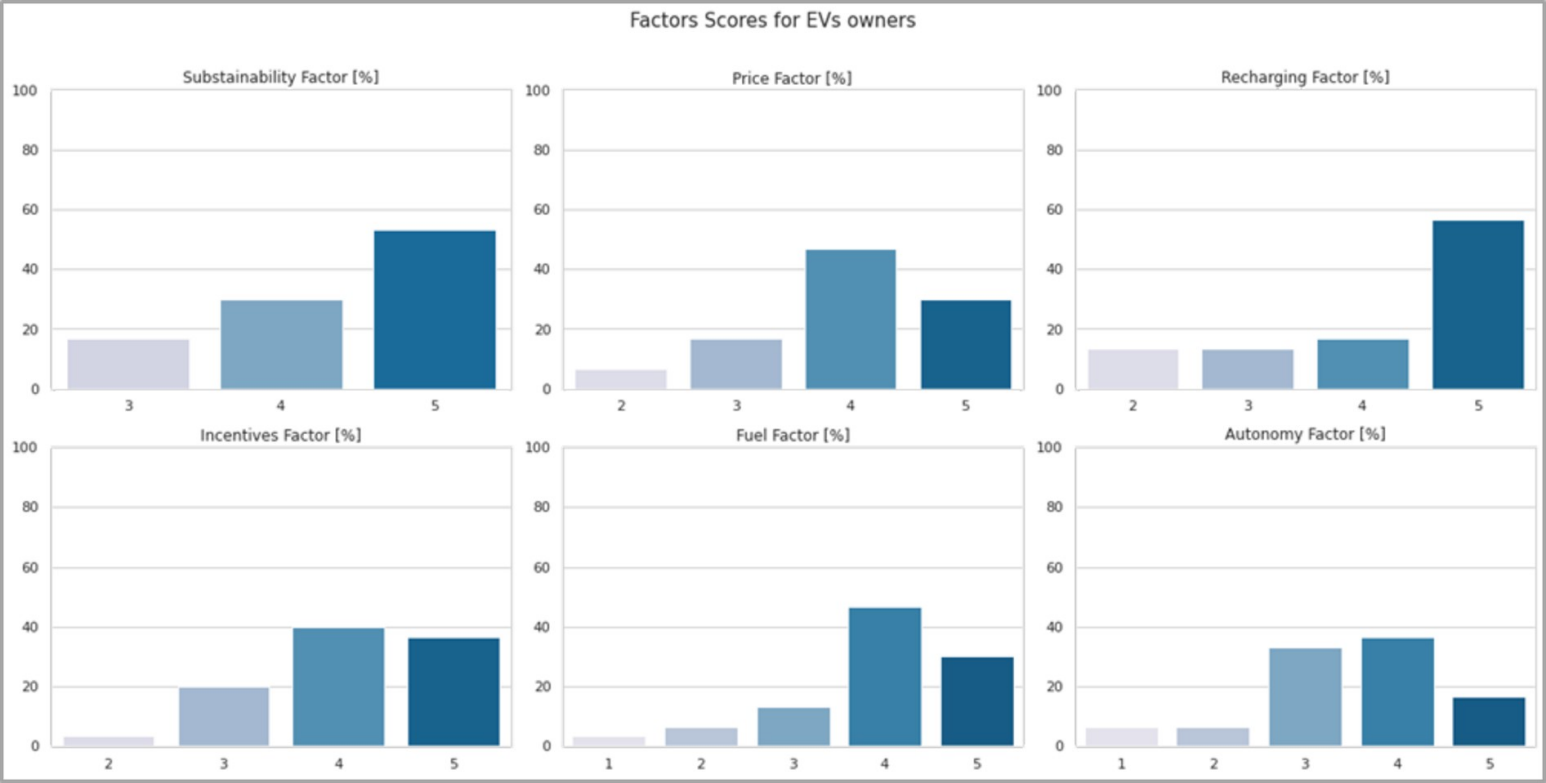
EVs owners’ opinion towards the main factors influencing the spread of EVs.

By looking at Fig 12, it is possible to derive some considerations.

- The *sustainability* factor could be included among those that were decisive for the choice, since the totality of EV owners provided a grade equal to or greater than 3.
- Similar reasoning applies to *incentives*, for which only one respondent believes that they were not important.
- Conversely, only a quarter of respondents provide a medium-low grade for the *price* factor.
- Most users are also satisfied with the savings on the cost of *fuel*.
- Finally, while the weights given to the vehicle’s *autonomy* factor are more widely distributed, there is clear confirmation from EVs owners that the availability of *charging points* is still an obstacle, since more than half attribute the maximum weight to this factor. This last consideration is confirmed in the answers given to the additional questions.

For this analysis, two different target variables were considered: the *current buying propensity* and the *future buying propensity*. In particular, "*future propensity*" means the purchase intentions that users will have starting from 2025, the year in which the experts estimate that the production costs of batteries will fall to such an extent as to make electric cars competitive with "traditional" ones in terms of price [35].

**Fig 13:**
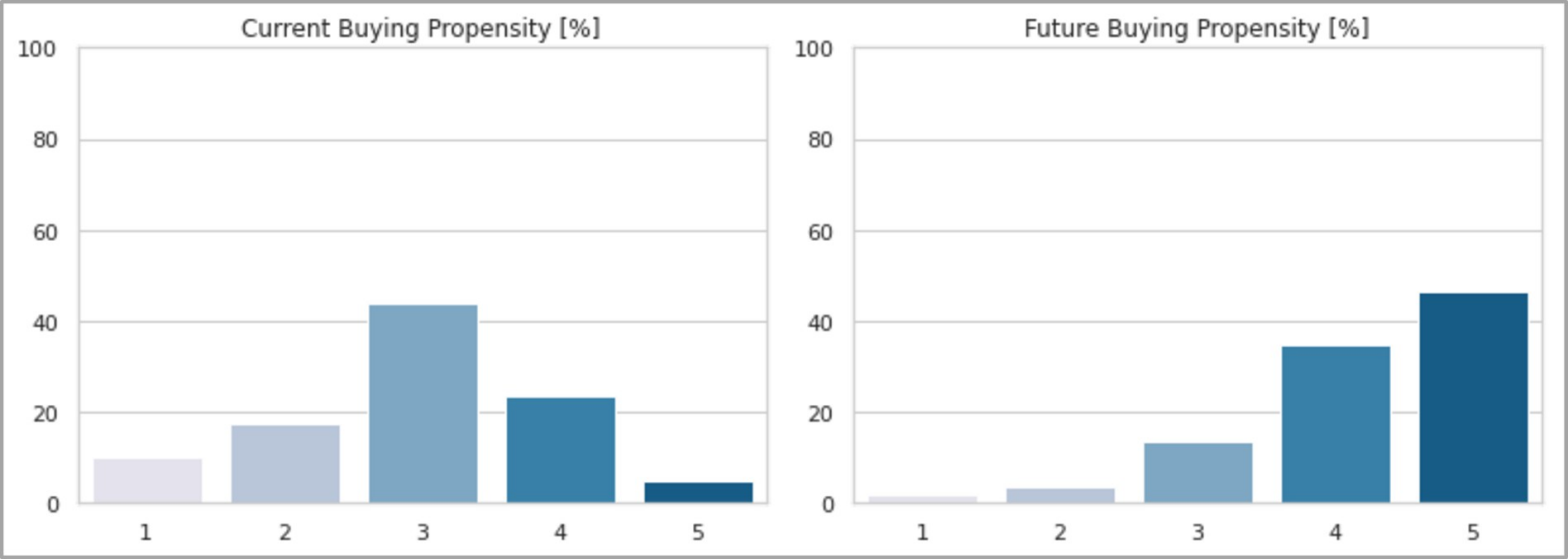
Current and future buying propensity of respondents.

As it can be seen from the two graphs in Fig 13 the clear change in respondents’ intentions is evident. Today, just under half of respondents (44%) have no clear opinion, with only 28.7% who would be willing to pay a higher price for the purchase and 27.3% who would have no intention of it. On the contrary, the scenario changes considerably regarding future prospects. In fact, the share of those who are inclined to buy an electric car rises to 80.7%, with 14.3% remaining indifferent and just 5% deciding not to buy it anyway. All this confirms once again that the price remains a binding factor to date.

We therefore proceeded defining predictive models able to estimate the *current* and *future* propensity to purchase of users. The same regressive models seen in the case of regional and provincial analysis were applied: *Linear Regression*, *Ridge Regressor*, *Decision Tree Regressor* and *Extreme Gradient Boosting Regressor*. However, it should be noted that in this case, unlike the previous ones, the numerical variable is discrete, not continuous. In fact, it can take as values 0,1 and 2, while the prediction made through one of the previous models returns continuous values in output. For this reason, the strategy used was to transform the continuous values obtained from the predictions into the discrete values assumed by the target variable according to special approximation criteria. In this way, it was then possible to use, as evaluation criteria, those that are generally used in the case of classification problems such as: *accuracy*, *precision* and *recall* score.

In order to assess model performances we implemented a K-Fold Cross Validation (CV) procedure. Based on this kind of technique, the original dataset is randomly partitioned into K equal sized subsamples. Of the K subsamples, a single subsample is retained as the validation data for testing the model, and the remaining K-1 subsamples are used as training data. In particular for the purpose of this study we implemented a 3-Fold CV procedure. Table 5 shows the weighted average scores± standard deviation for each K-Folds CV iteration of the model in the case of predicting *current propensity*.

**Table 5:** Weighted average scores for current propensity predictions over the 3 run of the CV procedure.

	Accuracy	Precision	Recall
**LR**	0.52 ± 0.04	0.57 ± 0.05	0.52 ± 0.04
**RR**	0.49 ± 0.04	0.62 ± 0.08	0.49 ± 0.04
**DT**	0.43 ± 0.02	0.44 ± 0.04	0.43 ± 0.03
**XGBR**	0.50 ± 0.10	0.53 ± 0.11	0.50 ± 0.10

The precision and recall metrics are computed for each label and the average is weighted by the support, that is the number of true instances of each label. Results are presented as: *value* ± *standard deviation*.

If compared with the performance of the models in case of the regional and provincial analysis, the difference between the LR and XGBR is negligible. The first one has an accuracy value of 0.52 that is slightly higher than the 0.5 accuracy of the XGBR.

For what concerns the prediction of *future propensity*, the main results are reported in Table 6. Also in this case a 3-folds CV procedure was applied to assess the robustness and model performances. We report in Table 6 the results of the weighted average of accuracy, precision and recall ± standard deviation.

**Table 6:** Weighted average scores for future propensity predictions.

	Accuracy	Precision	Recall
**LR**	0.79 ± 0.06	0.78 ± 0.05	0.79 ± 0.07
**RR**	0.78 ± 0.05	0.77 ± 0.04	0.78 ± 0.05
**DT**	0.76 ± 0.07	0.80 ± 0.06	0.76 ± 0.07
**XGBR**	0.80 ± 0.02	0.81 ± 0.04	0.80 ± 0.02

The precision and recall metrics are computed for each label and the average is weighted by the support, that is the number of true instances of each label. Results are presented as: *value* ± *standard deviation*.

The value of the accuracy is on average considerably higher than in the previous case. If for the prediction of the current propensity the accuracy value did not go beyond 0.52, in this case it reaches a maximum value of 0.8. This is due to the models that manage to estimate almost perfectly those who show a high propensity to buy and this increases the accuracy of the model, since these represent the greatest part of observations (almost 80%).

In addition to this, the good performance of the models in the case of the estimation of future propensity confirms what has already been seen in the case of regional and provincial analysis. As it has already been shown in Fig 3, the models were trained at time t and then tested at time t+1, providing good performance. Even in this case it is possible to apply the same reasoning. In fact, the implemented models are trained on the data collected today (2021) and they are much more performing in estimating the future propensity rather than the current one, as we have seen from the accuracy. By looking at the different models’ performance in Table 6, we can conclude that the XGBR is confirmed again as the most performing one, similarly to what happened for the national and regional case, even though we have to say that all of the 4 models performs quite similarly in the prediction tasks proposed. We also report the confusion matrices for the XGBR models for the current and future propensity in S.4 Section, S5 and S6 Figs in S1 File.

The XGBR model performed slightly worse than the LR model, only for the case of the current propensity prediction. Overall the 4 models showed robustness also in the cross validation performances and showed to be quite robust in the various cases considered.

### 4.4 The most important features

**Fig 14:**
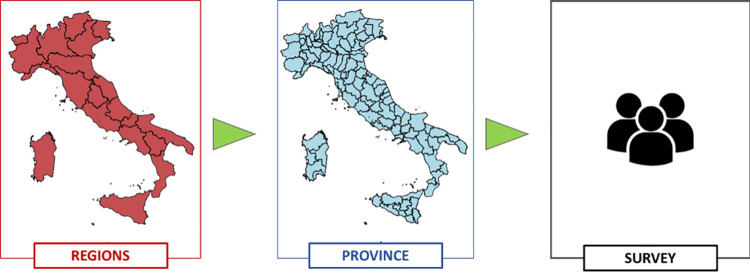
The workflow followed for the research. Starting from the regional analysis (red), the focus of the research was restricted to the province (blue), up to analyzing a sample of the population through the administration of a survey (black).

In our paper we described a novel three dimensional view of the Italian electric cars scenario. We summarize it graphically in Fig 14.

We can now proceed in the analysis of the common features which were found to be significant across the models.

Starting from the fact that in all three cases the *Extreme Gradient Boosting Regressor* gave the best results to be the best model, it is therefore possible to view the importance that each of the features has had in the definition of the model itself. The aspect that is really interesting is that in all three cases it is possible to identify some features that are the same or that at least refer to the same concept. The variables can be summarized per category as: number of charging points, the sustainability factors, and the availability of photovoltaic panels.

We can further analyze each of the these features:

- **PRICE:** with the exception of the provincial dataset, for which, due to the lack of data on online databases, it was not possible to obtain information on the average purchasing power of citizens, the price factor plays an important role in the defined predictive models. It has been included in the regional dataset in the form of Pro-Capite GDP (Gross Domestic Product), that is the average purchasing power of citizens, and represents the first variable in order of importance. It is not surprising that the regions with the highest percentages of circulating electric cars are those with the greatest purchasing power, a factor that confirms the weight of the price in the spread of these vehicles. In the case of the survey, on the other hand, the price factor was treated more explicitly, directly asking users to assign a weight to it. As already shown in Section 5.3, respondents confirmed the high importance of this factor.
- **RECHARGING POINTS**: regarding the regional and provincial analysis, it has already been highlighted above that the number of charging points is positively correlated with the number of electric cars. This in fact represents the main factor that weighs on the spread of electric cars and this has also been confirmed by the analysis of the data collected through the survey. In fact, the variable pdr_futuri indicates the propensity of users to buy an electric vehicle in the future when there will be greater road coverage of charging stations. About 80% were inclined, which also justifies the importance of the variable, thus confirming that this factor still represents an obstacle.
- **SUBSTAINABILITY:** the sustainability factor was explicitly covered in the case of the survey, where about 70% assigned it the maximum scores. Instead, in the case of the regional and provincial analysis, this factor has been linked to the variable indicating the number of exceedances of the limit threshold of PM10 dust (*number of excendacees of PM10*) and to the polluting potential (*polluting factor*). In fact, the data shows that the spread of electric cars is directly proportional to the first variable and inversely proportional to the second.
- **PHOTOVOLTAIC:** despite being in the low part of the ranking in order of importance, the feature that indicates the number of photovoltaic panels present in the region or province (*number of photovoltaic panels*) has a good correlation with the percentage of electric cars present in that region or province, as mentioned in Sections 4.1 and 4.2. The presence of a photovoltaic panel, as already expressed above, potentially plays a non-negligible role in convincing a consumer to buy an electric car. In fact, it allows users to recharge the battery of their car without incurring any cost, thanks to the fact that the energy used is the one produced through the photovoltaic panel. If in the case of the regional and provincial analysis this could only be a hypothesis, in the case of the survey analysis it has been confirmed. In fact, the percentage of respondents who would be inclined to buy an electric car goes from 43% to 78.4% if they have a photovoltaic panel in their home. In addition, about 59% of them would also be willing to install a private charging station to take full advantage of the recharge at no cost.

The description of the factors just exposed allows to have a clearer idea of what was the main objective of the study: to identify and understand the role of the main factors that influence the spread of electric mobility. The analyses carried out allow us to face the same study, but with three different points of view: the regional, the provincial and the individual one. Despite the necessary differences that do not allow to overlap the considerations made in the three cases, the just described concepts have allowed us to define what were the common factors for all three analyses, although with different forms and entities.

## 5. Conclusions

Predicting the electric vehicle scenario in Italy is an aspect of key importance for future electric network [15, 36]. This in fact could dramatically help in the definition of the future Italian electric network scenario and planning. Moreover the identification of factors which influence the purchase of electric cars, could help companies and governments in finding specific drivers to be promoted in the population to allow further diffusion of electric vehicles in the future.

In our work we implemented a machine learning pipeline to define a model capable of estimating the distribution of electric cars with high accuracy. The novelties of this study are manifold:

- we constructed a novel dataset crawling from public sources; we proceeded with the construction of such novel dataset after a thorough search in the literature in which we could identify the possible drivers in terms of electric mobility diffusion. Starting from them, the data were downloaded separately from the online databases and they were subsequently aggregated in forming two types of new datasets on which to implement the models. More precisely, a first analysis was carried out on a regional scale and later also on a provincial scale. For both, the target variable consists of the regional or provincial percentage of circulating electric cars. In both cases, the approach used to test the models was the same. In fact, they were trained at time t and then tested at time t+1, through the comparison between the predicted and real values. For this reason, in the construction phase of the datasets, data relating to five different years, from 2015 to 2019, were downloaded and aggregated.
- We designed and distributed a survey to test a sample of the population regarding electric mobility; The survey was novel and completely designed for the purpose of our study, showing the capability of testing the presence of similar factors identified both in the national, regional and local Italian dimensionality tested. Narrowing the field of research, the idea behind the survey was to find further confirmation regarding the main diffusion factors of electric mobility, already covered in previous analyses. In addition, it was possible to investigate those for which no data were available, such as economic incentives. By applying the same predictive models, the current and future buying propensity of users were estimated.
- We applied machine learning models and identification of important features for electric vehicles scenario prediction. The four machine learning models used were robust across the three dimensional datasets to further understand if similar factors were observable at a national, regional and sampled population scale. From the analysis on the local dimension we identified the same factors that can influence the diffusion of electric mobility in all three analyses, despite the different research scales. Both in the regional and provincial analysis, among the highlighted variables, all those that were linked to the main factors of diffusion of electric mobility were included, thus confirming the hypotheses made in the research phase. A further confirmation came from the analysis of the data collected through the survey, where the variables related to the factors again played a central role. More precisely, the price, the presence of charging infrastructure, the sustainability and the presence of photovoltaic panels emerged among those highlighted. All these factors had already been introduced in Section 1, based on the research done in the existing literature. The performed analyses have therefore made it possible to verify the hypotheses made in the research phase, finding the actual contribution that these factors have for the spread of electric mobility according to three different scales: regional, provincial and personal.All of this focusing on the Italian scenario, which, to the best of our knowledge, has not been previously analyzed as in this present thesis.

This study could certainly be extended to include new and interesting studies in the field. Novel approaches, for example are going towards the identification of machine learning models for solving practical traffic problems, which could be enlarged to include specific studies for electric cars [37], extending the study to second-hand and residual value estimation of electric vehicles [38], study of consumer-centric and multi-objective optimization frameworks based on the Energy-hub concepts [39] as well as adding polluting estimation data to cross-study together with the impact of electric cars in the environment [40].

Moreover the present study could be improved through the progressive updating of estimates based on new available data. It should be pointed out that the spread of electric mobility is an extremely recent phenomenon and for this reason the availability of data related to it is currently very limited. In addition, factors such as price, the presence of charging infrastructure and economic incentives could have a relevance that is limited mainly to the initial phase of deployment. In fact, as soon as the prices of the battery pack are lowered, the cost of the electric car will also decrease, and the incentives will certainly have less relevance.

Moreover, it is expected that in view of the estimates made by the PNRR, charging infrastructure will also have such a diffusion that it no longer represents an obstacle. On the basis of these considerations, it should be noted that this study therefore represents a starting point for the analysis of the phenomenon of Italian electric mobility, and it can be updated, focusing mainly on long-term factors, such as sustainability, which is certainly destined to cover an increasingly central role in coming years.

## 6. Ethical statement

Consultation for what concerns the ethical approval was requested to the CAREUS ethics committee of the University of Siena (https://en.unisi.it/research/ethics-committee-research-human-and-social-sciences-careus). The committee stated that given the questions of which the survey was composed of, which do not imply the presence of sensible and reserved information, the CAREUS did not need to express any ethical approval concerning the research hereby presented. Informed consent was obtained verbally by the distributor of the survey and a privacy statement included in the beginning of the survey. No minors were included in the survey study.

## Supporting information

S1 File This file containing the survey, survey data exploration and performance indicators description. (DOCX)
