# Supplementary material for: A machine learning approach to analyse and predict the electric cars scenario: The Italian case
Source: PLoS One. 2023 Jan 20;18(1):e0279040. doi: 10.1371/journal.pone.0279040 (PMC9858846; doi:10.1371/journal.pone.0279040)
Supplement: S1 File — (DOCX) [file pone.0279040.s001.docx]

**Supplementary Material**

**S.1: Description of the survey questions**

This paragraph lists the cover letter and the questions that compose the survey, which has been introduced in Section 2. The questions are ordered in the same way as they were presented to the respondents. Furthermore, they are grouped by the factors which they refer to. Since the survey was administered in Italian, both the presentation letter and the questions are translated in English.

**Cover letter**

*“Dear Participant,*

*By answering this short survey, whose completion time is only 3 minutes, you will provide a valuable contribution to my study.*

*Your answers will be valuable and if you choose to participate, I ask you to spend a few seconds to make sure you provide the answers that best describe what is requested.*

*Your answers will be anonymous, and the individual respondents will not be identified in any data or report in compliance with the GDPR privacy law 679/2016.*

*Thank you for your cooperation and for your time!”*

**The questions**

- **Screening questions**: *the aim of these first questions is divide the respondents on the basis of the interest in the topic.*

1. Do you have driving license?
2. Yes
3. No
4. Are you interested in electric mobility?
5. Yes
6. No

- **Cars fleet questions**: *the aim is to collect statistical information related to the cars owned by the respondents.*

1. How many cars do you own?
2. 0
3. 1
4. 2
5. 3 or more
6. How many cars does your family own?
7. 0
8. 1
9. 2
10. 3 or more
11. How many of these have Euro 3 emission class (registered before 01/01/2006)?
12. 0
13. 1
14. 2
15. 3 or more
16. How many of these have Euro 34 emission class (registered before 01/01/2011)?
17. 0
18. 1
19. 2
20. 3 or more
21. How many of these have Euro 5 emission class (registered before 01/01/2015)?
22. 0
23. 1
24. 2
25. 3 or more
26. How many of these have Euro 3 emission class (registered after 01/01/2015)?
27. 0
28. 1
29. 2
30. 3 or more

1. How many of these are powered by gasoline?
2. 0
3. 1
4. 2
5. 3 or more
6. How many of these are powered by diesel?
7. 0
8. 1
9. 2
10. 3 or more
11. How many of these are powered by methane?
12. 0
13. 1
14. 2
15. 3 or more
16. How many of these are hybrid or full electric?
17. 0
18. 1
19. 2
20. 3 or more

- **Habits with the car**: *these questions aim to investigate users' habits in the general use of the car. Users with higher usage could potentially be the most likely to change it in favor of an electric or hybrid vehicle.*

1. On a scale between 1 (very rarely) and 5 (very often), how often do you use the car?
2. 1
3. 2
4. 3
5. 4
6. 5
7. What is the main reason why you use the car?
8. Job/Study
9. Leisure
10. Travel
11. Generic displacements (i.e. Shopping)
12. How many km do you travel on average in a year?
13. Less than 10.000 km
14. Between 10.000 and 20.000 km
15. More than 20.000 km
16. On a scale from 1 (very rarely = more than 10 years) to 5 (very often = every 1-2 years), how often do you change cars?
17. 1
18. 2
19. 3
20. 4
21. 5
22. How much are you willing to pay for a new car?
23. Up to 10.000€
24. 10.000 – 20.000 €
25. 20.000 – 30.000 €
26. More than 30.000 €

- **Habits of electric cars owner**: *these types of questions are only given to those who answer that they own at least 1 Electric or Hybrid car in question 12. The aim is to investigate the reasons that led to the purchase of an electric / hybrid car and to analyze the related habits.*

1. Since you own an Electric/Hybrid car, is it a company or private car?
2. Company car
3. Private car
4. What is its autonomy in terms of km?
5. Less than 100 km
6. 100 – 200 km
7. 200 – 400 km
8. More than 400 km
9. On a scale between 1 (very rarely) and 5 (very often), how often do you recharge the car battery?
10. 1
11. 2
12. 3
13. 4
14. 5
15. Where are you used to recharge the battery?
16. Private charging infrastructure
17. Public charging infrastructure
18. Company’s charging infrastructure
19. For what concerns the aspects related to the electrical component of your car (Ex: autonomy, performance, charging ...), are you satisfied with the purchase?
20. Yes
21. No

- **Electric mobility factors**: with this type of questions, we want to analyze the perception that people have with respect to the main drivers related to the diffusion of electric cars.

1. On a scale from 1 (not at all important) to 5 (very important), how important is the factor of ENVIRONMENTAL SUSTAINABILITY in the choice of car?
2. 1
3. 2
4. 3
5. 4
6. 5
7. On a scale from 1 (not at all) to 5 (a lot), how much does the PRICE for buying an electric/hybrid car currently weigh?
8. 1
9. 2
10. 3
11. 4
12. 5
13. On a scale from 1 (not at all) to 5 (a lot), how much does the availability of public battery CHARGING POINTS currently weigh for the purchase of an electric/hybrid car?
14. 1
15. 2
16. 3
17. 4
18. 5
19. On a scale from 1 (not at all) to 5 (a lot), how much does the presence of national or regional INCENTIVES currently weigh for the purchase of an electric/hybrid car?
20. 1
21. 2
22. 3
23. 4
24. 5
25. On a scale from 1 (not at all) to 5 (a lot), how much does the FUEL SAVINGS weigh for the purchase of an electric/hybrid car?
26. 1
27. 2
28. 3
29. 4
30. 5
31. Considering that currently the average range of batteries for electric cars is about 250 km, on a scale from 1 (not at all) to 5 (a lot), how much is enough given your needs?
32. 1
33. 2
34. 3
35. 4
36. 5

- **Purchase propensity**: *with these questions we want to analyze how purchase intentions change in the future.*

1. Currently the cost of electric cars is on average higher than the one of traditional cars. On a scale from 1 (not at all inclined) to 5 (very inclined), how much would you be inclined to pay a higher price for a car in its hybrid/electric version?
2. 1
3. 2
4. 3
5. 4
6. 5
7. It is estimated that for 2025, thanks also to the drop in battery production costs, the price gap between hybrid/electric cars and traditional ones will be minimized. On a scale of 1 (not at all) to 5 (a lot), how much would it favor the purchase of an electric car?
8. 1
9. 2
10. 3
11. 4
12. 5
13. The number of charging points is in a phase of strong growth, on a scale from 1 (not at all) to 5 (a lot), how much does it contribute to the purchase of an electric car in the coming years?
14. 1
15. 2
16. 3
17. 4
18. 5

- **Place of residence**: *the aim is to collect some statistical information related to the geographical area of residence.*

1. In which region do you live?

*Select the region*

1. In which province do you live?

*Select the province*

1. Where do you live?
2. City center
3. Suburbs
4. Out of town
5. On a scale from 1 (not at all necessary) to 5 (very necessary), how much do you think public transport is necessary for your usual displacements?
6. 1
7. 2
8. 3
9. 4
10. 5

- **Photovoltaic panels**: *the aim is to analyze the intentions of users to purchase an electric/hybrid car in parallel with the use of photovoltaic panels that would allow charging at no cost.*

1. Do you have photovoltaic panels in your home?
2. Yes
3. No
4. On a scale from 1 (not at all inclined) to 5 (very inclined), how inclined would you be to buy an electric/hybrid car while NOT having photovoltaic panels in your home?
5. 1
6. 2
7. 3
8. 4
9. 5
10. On a scale from 1 (not at all inclined) to 5 (very inclined), how inclined would you be to buy an electric/hybrid car having photovoltaic panels in your home that would allow charging at no cost?
11. 1
12. 2
13. 3
14. 4
15. 5
16. Do you have a private garage/parking for the car?
17. Yes
18. No
19. If you have an electric/hybrid car and a private garage/parking, on a scale from 1 (not at all inclined) to 5 (very inclined), how inclined would you be to invest in the installation of a private charging station?
20. 1
21. 2
22. 3
23. 4
24. 5

- **Personal questions**: *the aim is to collect some personal information for statistical purposes*.

1. What is the number of members of your family?
2. 1
3. 2
4. 3
5. 4
6. 5 or more
7. What is your age group?
8. 18-25
9. 25-50
10. 50-75
11. 75+
12. What is your gender?
13. Male
14. Female
15. Not Specified
16. What is your profession?
17. Entrepreneur/ Freelancer
18. Employee
19. Executive
20. Worker
21. Farmer/ Merchant/ Craftsman
22. Retired
23. Unemployed
24. Student

### S.2: Survey Data Statistical Exploration

The number of respondents is 300, each of whom answered the questions that compose the survey. As a result, the data containing the users' responses have been downloaded and then imported into Google Colab in order to extract as much information as possible and define a prediction model. The final survey dataset is therefore composed of 300 rows, each of which corresponds to a user and 44 features, which represent the information collected through the survey questions. Let us now describe the main results obtained. First, it is not surprising that more than 90% of respondents showed an interest in electric mobility. This aspect is a first confirmation of the increasing awareness of people towards a more sustainable mobility, as mentioned in Section 1. In fact, only a small part, more precisely 7.2% which corresponds to 21 people, answered negatively to the question in which they were asked about their interest in this topic. The same people then confirmed their position on the subject by showing a low propensity to buy an electric vehicle, as it is shown in Figure S1. In fact, regarding the "Future Buying Propensity" the percentage of those who are disinterested remains high at around 28.5%. This value is much higher when compared with the percentages relating to the total number of respondents, where the disinterested stand at just 5.1%.


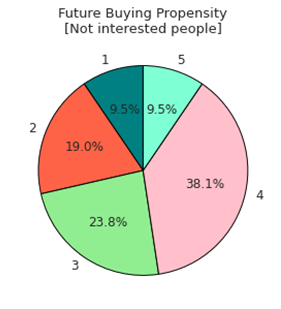


**Figure** **S1** - Future buying propensity of disinterested respondents

Referring again to the total number of respondents, the main statistical information on them is graphically shown in Figure S2.


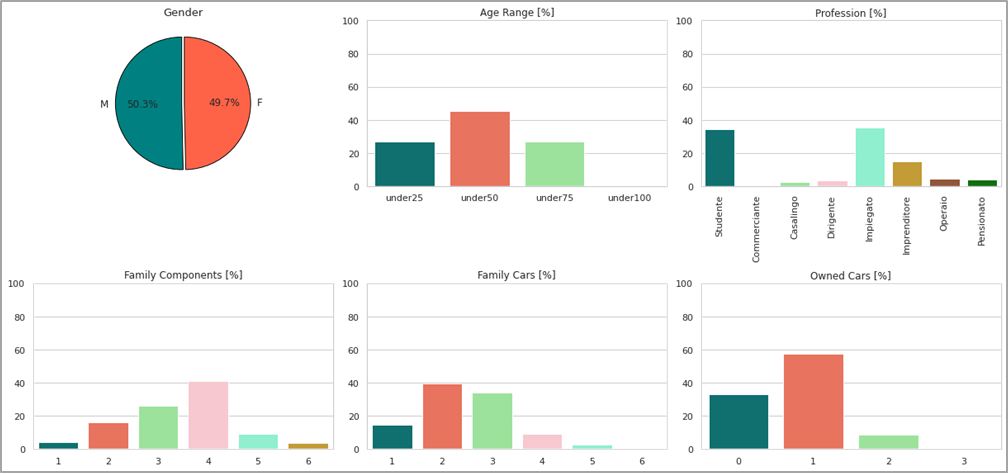


**Figure S2** – Main statistical information on Survey’s respondents. Starting from the top-left corner, it shows the distribution of the respondents in terms of: *Gender, Age Range, Profession, Number of Family’s components, Number of Family’s cars, Number of owned cars.*

As it can be noticed, the respondents are perfectly balanced from the point of view of gender, while more than 40% of them are people aged between 25 and 50 years with the under 25 and the 50-75 group who share the remaining 60% almost equally. More than half belong to large families consisting of at least three members. This aspect is also reflected in the total number of cars owned by the families themselves, where only about 18% of respondents own a single vehicle for the whole family. Remaining on the statistics relating to cars, the graphs included in Figure S3 show the percentages in terms of emission class and type of power supply. In particular, the values shown in the x-axis of the graphs in Figure S3 indicate the number of cars owned by the respondent that correspond to that type of power supply or that emission class.


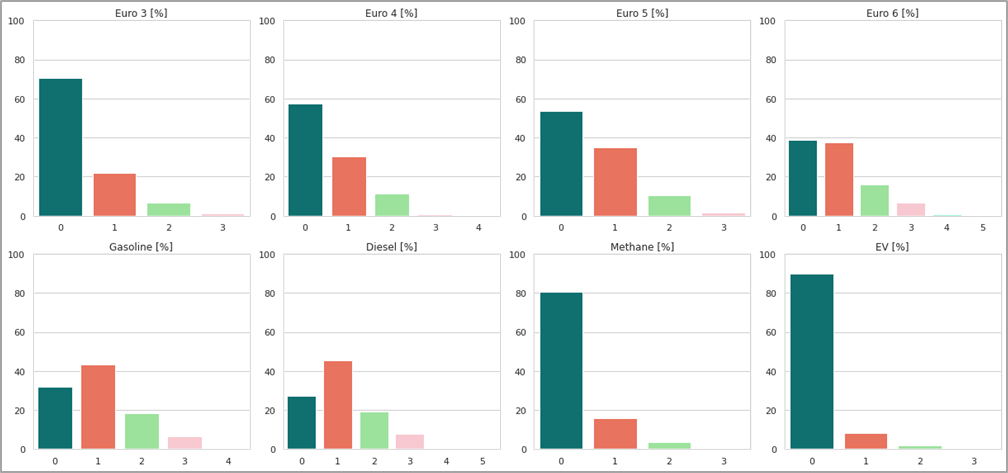


**Figure S3** – Main statistical information on cars’ emission classes and power supply. On the x-axis the number of owned cars of that emission class or power supply is reported. The y-axis reports the percentage of users for which the number of owned cars of that emission class or power supply is the one reported on the x-axis.

From the point of view of the type of power supply, the great majority of users answered that they do not own low-emission cars. In particular, by looking at the graph for electric cars, just 10% of the participants answered that they owned at least one. For what concerns the emission class, they seem to be more balanced.

The pie charts shown in Figure S4 highlight the percentages related to the emission class and the type of power supply considering not the individual respondents, but the total of cars obtained by adding the number of cars owned by each individual respondent.


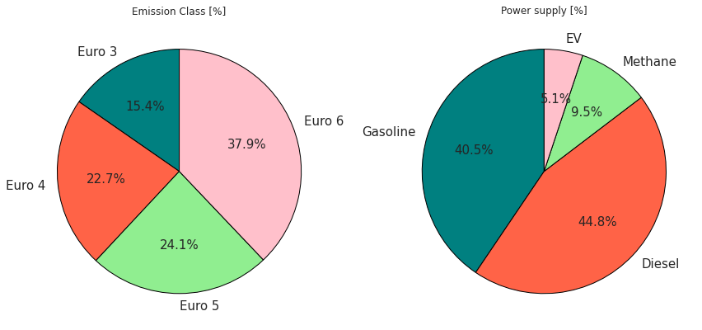


**Figure S4** – Considering the total number of cars computed as the sum of the cars owned by each respondent, the pie charts illustrate the fractions of them in terms of emission classes and power supply.

As it is possible to notice, the low-emission cars are just less than 15% of the total. Diesel and gasoline cars are balanced. On the contrary, looking at the emission classes, most of the respondents have certainly replaced the car in the last ten years. Almost 62% of them, in fact, own cars with the most recent emission classes, with the Euro 6 one exceeding the share of Euro 5. The most polluting cars still represent a substantial percentage (almost 38%).

We now report statistical analysis for what concerns users' car habits, as shown in Figure S5.


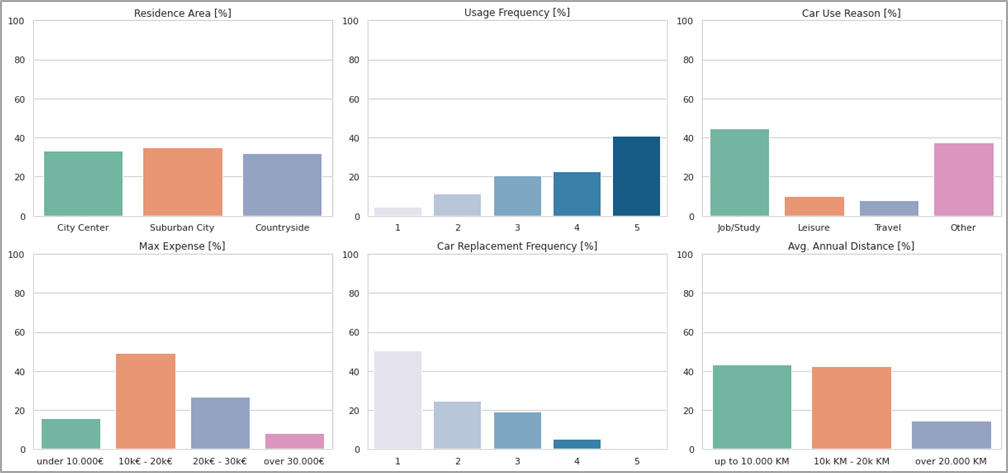


**Figure S5** – Main statistical information related to users’ habits with the car. Starting from the top-left corner, it shows the percentage of users grouped by *Residence Area*, the *Usage Frequency* of the car, the *Main Reason* to use the car, the *Max Expense* to replace the car, the *Replacement Frequency* and the *Average Annual Distance*.

Although respondents are equally distributed among the three types of areas of residence, approximately 60% of them use the car frequently. Travel is mainly motivated by work or study (45%), immediately followed by generic reasons such as going shopping (about 38%). Despite the high frequency of use of the car, about 15% of the users travel more than 20 thousand Km a year, while the remaining are equally divided by the other two lower bands (up to 10.000 km and 10-20 thousand km). Finally, for what concerns the replacement of the old car with a new one, about half of the respondents tend to keep their car for more than 10 years and more than 60% are not planning to spend more than € 20.000 on it.

**S.3: Performance Indicators**

- Equation **S1**:

|  | $RMSE=\sqrt{\frac{1}{n}\sum_{i}^{n} \left( \hat{y}_{i}-y_{i} \right)^{2}}$ | (S1) |
| --- | --- | --- |

- Equation **S2**, where $\underline{y}$ is the mean value of the observed data:

|  | $R^{2}=1-\frac{\sum_{i}^{n} \left( y_{i}-\hat{y}_{i} \right)^{2}}{\sum_{i}^{n} \left( y_{i}-\underline{y} \right)^{2}}$ | (S2) |
| --- | --- | --- |

###
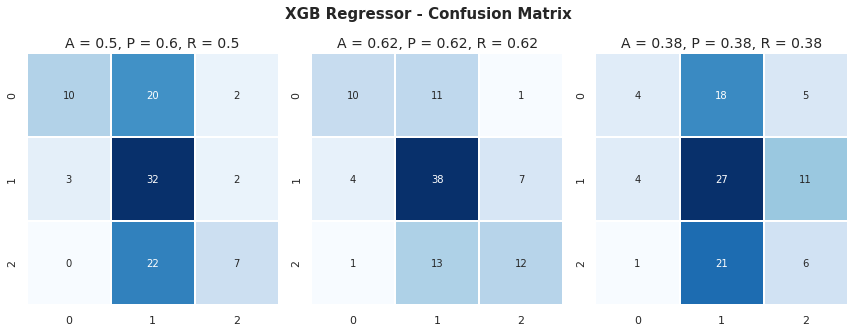
S.4: Confusion Matrices for the Survey Dataset Experiments

**Figure S6** - XGBR Confusion Matrices for current propensity predictions. The confusion matrices refer to each implementation of the K-Fold Cross Validation. In addition, the main scores for every implementation are reported. “A” is the accuracy, “P” the precision and “R” the recall.


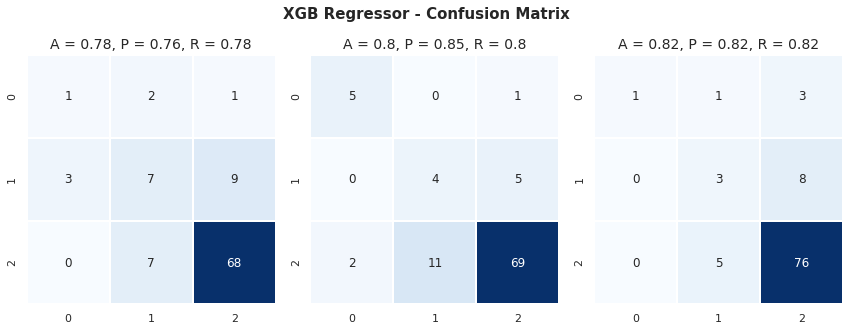


**Figure S7** - XGBR Confusion Matrices for future propensity predictions. The confusion matrices refer to each implementation of the K-Fold Cross Validation. In addition, the main scores for every implementation are reported. “A” is the accuracy, “P” the precision and “R” the recall.
